# Proximal tibial trabecular bone mineral density is related to pain in patients with osteoarthritis

**DOI:** 10.1186/s13075-017-1415-9

**Published:** 2017-09-12

**Authors:** Wadena D. Burnett, Saija A. Kontulainen, Christine E. McLennan, Diane Hazel, Carl Talmo, David R. Wilson, David J. Hunter, James D. Johnston

**Affiliations:** 1 University of Saskatchewan, 57 Campus Drive, Saskatoon, SK S7N 5A9 Canada; 2 New England Baptist Hospital, Boston, MA USA; 3 University of British Columbia, Vancouver, BC Canada; 4 University of Sydney, Sydney, NSW Australia

**Keywords:** Osteoarthritis, Bone mineral density, Tibia, Pain, Computed tomography

## Abstract

**Background:**

Our objective was to examine the relationships between proximal tibial trabecular (epiphyseal and metaphyseal) bone mineral density (BMD) and osteoarthritis (OA)-related pain in patients with severe knee OA.

**Methods:**

The knee was scanned preoperatively using quantitative computed tomography (QCT) in 42 patients undergoing knee arthroplasty. OA severity was classified using radiographic Kellgren-Lawrence scoring and pain was measured using the pain subsection of the Western Ontario and McMaster Universities Arthritis Index (WOMAC). We used three-dimensional image processing techniques to assess tibial epiphyseal trabecular BMD between the epiphyseal line and 7.5 mm from the subchondral surface and tibial metaphyseal trabecular BMD 10 mm distal from the epiphyseal line. Regional analysis included the total epiphyseal and metaphyseal region, and the medial and lateral epiphyseal compartments. The association between total WOMAC pain scores and BMD measurements was assessed using hierarchical multiple regression with age, sex, and body mass index (BMI) as covariates. Statistical significance was set at *p* < 0.05.

**Results:**

Total WOMAC pain was associated with total epiphyseal BMD adjusted for age, sex, and BMI (*p* = 0.013) and total metaphyseal BMD (*p* = 0.017). Regionally, total WOMAC pain was associated with medial epiphyseal BMD adjusted for age, sex, and BMI (*p* = 0.006).

**Conclusion:**

These findings suggest that low proximal tibial trabecular BMD may have a role in OA-related pain pathogenesis.

**Electronic supplementary material:**

The online version of this article (doi:10.1186/s13075-017-1415-9) contains supplementary material, which is available to authorized users.

## Background

Knee osteoarthritis (OA) is a debilitating and painful disease characterized by changes in cartilage and subchondral bone. Pain is a complex combination of social, psychological and biological factors [1], and is often the primary sign that a patient may be afflicted with OA [2]. Unfortunately, the local biological pain pathogenesis within the knee joint is poorly understood [3] as it could be related to many structural factors (e.g., altered joint alignment [4], bone marrow lesions (BMLs) [5], or cysts [6]). Knee OA is commonly characterized by altered subchondral properties, including altered subchondral bone thickness [7], bone volume fraction [8], and volumetric bone mineral density (BMD) [9]. Importantly, altered BMD may disrupt local innervation [10] and/or the local mechanical behavior of bone [11], and thus may be a factor in OA-related knee pain.

To date, research investigating association between OA-related knee pain and bone has focused primarily on bone near the subchondral surface (e.g., subchondral cortical and subchondral trabecular bone) [12, 13]. Adjacent trabecular bone (e.g., epiphyseal bone, metaphyseal bone) is also affected by OA [9], with observations of thinner trabeculae, lower bone volume fraction, and lower density with progressing OA severity [14–16]. To date, there are no studies reporting relationships between epiphyseal or metaphyseal trabecular BMD and pain. A recent finite element (FE) study conducted by Amini et al. [17] has suggested that low epiphyseal trabecular bone density in OA [14–16], which is directly linked to the elastic modulus of epiphyseal bone [18], may explain OA proximal tibiae being less stiff than normal [17]. Importantly, a less stiff proximal tibia would result in higher bone deformation potentially explaining (at least to some degree) OA-related knee pain.

A clear understanding of pain pathogenesis is crucial for rational therapeutic targeting [19]. Further, as pain is the reason patients seek medical care, rational treatment targeting requires specific understanding of which structures contribute to pain [19]. With the aim of furthering our understanding of potential factors that may influence knee pain, the objective of this study was to investigate relationships between proximal tibial epiphyseal and metaphyseal trabecular BMD and OA-related knee pain.

## Methods

### Study participants

In total 42 participants with OA were recruited prior to total knee replacement (TKR) (17 male, 25 female; mean age 64, SD ± 10.1 years; mean body mass index (BMI) 28.7 ± 3.7; 18 left, 24 right) [13]. Study exclusion criteria included pregnant women, patients having a revision replacement instead of primary knee replacement, and patients with a prior history of bone pathologic change at the knee joint. The Institutional Research Board of the New England Baptist Hospital approved the study. Informed consent was obtained from all study participants.

### Participant assessment

OA severity was classified using Kellgren-Lawrence (KL) scoring [20]; participants had severity scores of 2–4. Pain severity was measured at the affected knee joint using the pain subsection of the Western Ontario McMasters Osteoarthritis Index (WOMAC) [21]. Participants were asked to assess the level of pain in the affected knee joint within the past 24 hours while walking on a flat surface, going up or down stairs, nocturnal pain at night in bed, sitting or lying down, and standing upright using a 5-point Likert scale (0–4). Individual element pain scores were then summed for a possible WOMAC pain score of 20. Summed pain scores ranged from 4 to 16. We also used the Self-Administered Comorbidity Questionnaire [22] to assess participants for any potential confounding comorbidities (e.g., diabetes mellitus or heart disease).

### Computed tomography (CT) scan acquisition

We used a single-energy clinical CT scanner (Lightspeed 4-slice, General Electric, Milwaukee, WI, USA) for bone imaging. A solid quantitative CT (QCT) reference phantom of known bone mineral densities (Model 3 T, Mindways Software Inc, Austin, TX, USA) was placed under the participants and included in all CT scans. Participants were oriented supine within the CT gantry and both legs were simultaneously scanned. Scans included the distal femur, patella, proximal tibia, and the 66% tibial shaft site proximal to the distal tibial endplate [23]. Only the proximal tibia and the 66% tibial shaft site were used in the current analysis.

CT scanning parameters included: 120 kVp tube voltage; 150 mAs tube current-time product; axial scanning plane; 0.625-mm isotropic voxel size (0.625 slice thickness, 0.625 mm × 0.625 mm in-plane pixel size); ~ 250 slices; and ~ 60s scan time. A standard bone kernel (BONE) was used for CT image post-processing. The effective radiation dose was ~ 0.073 mSv per scan, estimated using shareware software (CT-DOSE, National Board of Health, Herley, Denmark). For comparison, the average effective radiation dose during a transatlantic flight from Europe to North America is about 0.05 mSv [24].

### CT image analysis

We used a custom algorithm developed specifically for this study (Matlab 2010b; MathWorks, Natick, MA, USA), combined with manual segmentation to determine epiphyseal and metaphyseal trabecular BMD. We considered the epiphyseal region (subarticular region) as the proximal tibial volume between the subchondral surface and the epiphyseal line [25]. A single user (WDB) performed all segmentations and analyses. As this algorithm was developed specifically for this study, we assessed repeatability in a precision study performed on an independent sample of healthy participants and participants with OA [26] using recommended methods [27]. In summary, 14 participants were scanned three times with repositioning between each scan (42 scans, 28 degrees of freedom (DOF)). The repeatability expressed as precision error, of each BMD measurement was assessed using root mean square coefficients of variation (CV%) and ranged from 0.7% to 3.6%.

**Fig. 1:**
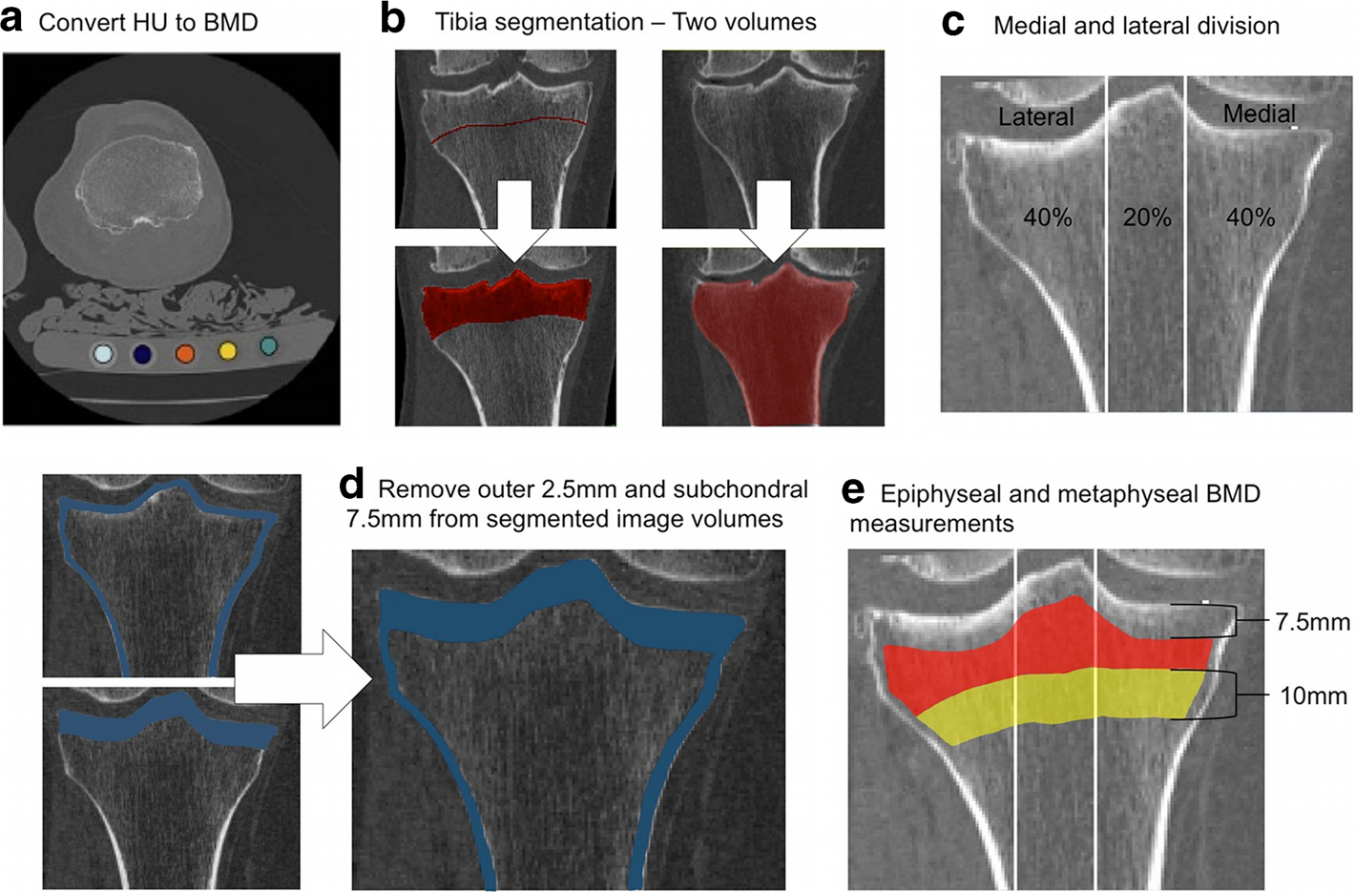
Methodological process consists of converting computed tomography (CT) grayscale intensities to bone mineral density (BMD) using a quantitative CT (QCT) reference phantom (**a**), followed by building two imaged volumes for each tibia, one with manual correction at the epiphyseal line and one using the full tibia (**b**). Imaged volumes were divided into lateral and medial regions (**c**) and then the outer 2.5-mm and subchondral 7.5-mm depth were removed from each imaged volume (**d**). BMD measurements included epiphyseal BMD between the epiphyseal line and 7.5 mm from the subchondral surface and metaphyseal BMD 10 mm distal from the epiphyseal line (**e**)

To derive BMD, grayscale Hounsfield units (HU) were converted to equivalent volumetric BMD (mg/cm^3^ K_2_HPO_4_) using subject-specific linear regression equations developed from known densities ranging from − 50 to 375 mg/cm^3^ K_2_HPO_4_ within the QCT phantom included in each individual axial image (*r* ^2^ > 0.99) [28] and interpolation to determine equivalent volumetric BMD values. Higher density values were linearly extrapolated (Fig. 1a). Subject-specific half maximum height thresholds [29] were then determined to define the proximal tibial subchondral and cortical surfaces. Two 3D image volumes were built, one including the entire proximal tibia as previously described [13, 28] and another by segmenting individual serial images using semi-automatic region growing and manual correction at the epiphyseal line (Fig. 1b). Both sets of imaged volumes were segmented using commercial software (Analyze10.0; Mayo Foundation, Rochester, MN, USA) and an interactive touch-screen tablet (Cintiq 21UX; Wacom, Krefeld, Germany). Imaged volumes were reoriented to a neutral position where medial and lateral plateaus were approximately parallel. We then divided the imaged volumes into medial and lateral compartments, measured by using 40% of the maximum medial-lateral axis of each respective side [16] (Fig. 1c).

To ensure that trabecular BMD measurements did not include cysts (which would lead to arbitrarily low measures of BMD) [13, 30] or peripheral high-density cortical bone, the most proximal 7.5-mm region (relative to the subchondral surface) was removed from the segmentations (Fig. 1d), as was 2.5 mm of peripheral cortical bone (Fig. 1d). The 7.5-mm depth was based upon observed cyst locations from our earlier work [13, 30] and work by Chiba et al. [31], which limited depth analyses to 5 mm from the subchondral surface. In extreme cases, large cysts extended from the subchondral cortical region (0 − 2.5 mm) through the subchondral trabecular region (2.5–5 mm) and occasionally into depths greater than 5 mm from the subchondral surface. By using a conservative 7.5-mm depth from the subchondral surface, we ensured the exclusion of large cysts from our analysis. Following material removal, we measured epiphyseal trabecular BMD from the 7.5-mm depth to the epiphyseal line (Fig. 1e), which was located approximately 15 mm from the subchondral surface. Metaphyseal trabecular BMD was measured 10 mm distal to the epiphyseal line (Fig. 1e).

We included cortical BMD of the tibial shaft (66% of the tibial length, proximal from the distal tibial plateau) [23] to assess whether associations with pain were systemic or joint-specific. More specifically, if similar associations between pain and BMD were observed at the proximal tibia and tibial shaft, this would indicate systemic effects with low BMD being a plausible secondary effect of other factors, such as mechanical loading, nutrition or medication [32]. Tibial shaft cortical BMD was segmented using subject-specific half-maximum-height thresholds, and measured using commercial software (Analyze10.0; Mayo Foundation, Rochester, MN, USA).

### Statistical analysis

We first checked all underlying assumptions for multiple linear regression (assumptions of linear relationships, homoscedasticity, independency and normality of residuals) using standardized residual scatter plots, P-P plots, and histograms [33]. We identified any outliers using the modified Thompson tau (τ) test [34].

We report univariate correlation coefficients (Pearson) between pain, BMD, age, sex, and BMI and illustrate associations between pain and BMD with scatter plots and coefficients of determination (*R* ^*2*^) from linear regression. We used hierarchical multiple linear regression analyses to explain the variance in total WOMAC pain. We selected age, sex, and BMI as covariates for our base model based on observed correlation in univariate analysis (age and WOMAC pain) and literature (age, sex, and BMI) evaluating relationships between BMD and pain [12, 35]. All BMD measurements (total and regional epiphyseal BMD, total metaphyseal BMD, and tibial shaft cortical BMD) were individually added to our base model. We assessed multicollinearity between all independent variables in each model using variance inflation factor (VIF), setting the maximum tolerance value as 10. We report adjusted *R* ^2^, change in *R* ^2^ from the base model (Δ), standardized beta (β)-coefficients, and *p* values. Statistical significance was defined as *p* < 0.05, and analyses were performed using SPSS 21.0 (IBM, Armonk, NY, USA).

## Results

**Fig. 2:**
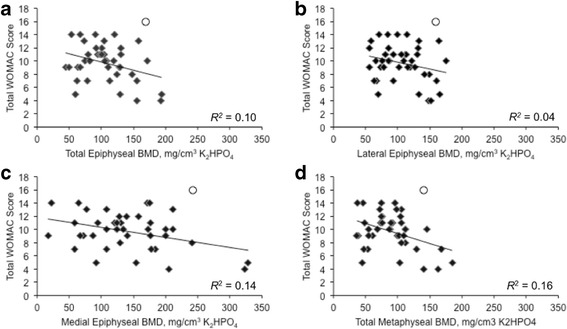
Scatter plots and coefficients of determination (*R* ^*2*^) of the unadjusted relationships between total Western Ontario and McMaster Universities Osteoarthritis Index (WOMAC) score and total epiphyseal bone mineral density (BMD) (*p* = 0.040) (**a**), lateral epiphyseal BMD (*p* = 0.187) (**b**), medial epiphyseal BMD (*p* = 0.015) (**c**), and total metaphyseal BMD (*p* < 0.009) (**d**). The single outlier is noted as a circle, and was not included in the bivariate analysis

Characteristics of all study participants, including age, sex, BMI, KL grades, joint space narrowing (JSN) score, non-weight-bearing alignment scores, and BMD measurements are shown in Table 1. As per the modified Thompson τ test [34], we identified a single outlier based on the total WOMAC pain score with a τ value outside of the sample’s rejection zone (τ > 5.56), and removed it from the analysis. All underlying assumptions for linear regression were appropriately met. There was no evidence of multicollinearity between independent variables in any of our models. Unadjusted relationships between total WOMAC pain and total or regional epiphyseal or metaphyseal BMD measurements are presented in Fig. 2. Pearson correlation analyses in all participants, and in male and female patients are presented in Additional files 1, 2 and 3: Tables S1–S3.

**Table 1:** Descriptive statistics for background characteristics of study participants

Characteristic	Without outlier
Age (mean ± SD)	64.1 ± 10.2
Sex (male:female)	17:24
BMI (mean ± SD)	28.6 ± 3.7
Side (left:right)	17:24
OA severity (KL) (0/1/2/3/4)	0/0/2/21/18
WOMAC score	9.7 ± 2.8
Medial joint space narrowing (0/1/2/3)	0/6/9/24^a^
Lateral joint space narrowing (0/1/2/3)	30/5/0/4^a^
Non-weight-bearing alignment	27 varus, 6 neutral, 8 valgus
Total epiphyseal BMD, mg/cm^3^ K_2_HPO_4_ (mean ± SD)	106 ± 37
Lateral epiphyseal BMD, mg/cm^3^ K_2_HPO_4_ (mean ± SD)	106 ± 34
Medial epiphyseal BMD, mg/cm^3^ K_2_HPO_4_ (mean ± SD)	141 ± 68
Total metaphyseal BMD, mg/cm^3^ K_2_HPO_4_ (mean ± SD)	90 ± 36

*BMI* body mass index, *OA* osteoarthritis, *KL* Kellgren-Lawrence grade, *WOMAC* Western Ontario and McMaster Universities Osteoarthritis Index, *BMD* bone mineral density

^a^Joint space narrowing scores not available in 2 participants

Regression models predicting variance in pain are presented in Table 2. After adding total epiphyseal BMD to the base model, (of age, sex, and BMI) the coefficient of determination (*R* ^2^) for total pain improved (Δ*R* ^2^ = 0.12). Our models improved when medial epiphyseal BMD (Δ*R* ^2^ = 0.15) and metaphyseal BMD (Δ*R* ^2^ = 0.12) were independently added to our base model. There was no association between cortical BMD at the 66% tibial site and pain.

**Table 2:** Adjusted coefficients of determination (*R* ^*2*^), standardized beta coefficients (β), and level of significance (*p*) of the base model (age, sex, and BMI) and change in the base model *R* ^*2*^ (Δ) when including bone mineral density (BMD) at the total and regional proximal tibia to predict variance in total WOMAC pain

		Total WOMAC		
		R^2^	β	*p* value
Base model		**0.16**		**0.023**
	Age		**−0.41**	**0.011**
	Sex		0.19	0.206
	BMI		0.12	0.448
Total epiphyseal		**0.28**		**0.003**
	Δ	0.12		**0.013**
	Age		**−0.41**	**0.007**
	Sex		0.08	0.596
	BMI		0.18	0.234
	BMD		**−0.38**	**0.013**
Lateral epiphyseal		**0.21**		**0.014**
	Δ	0.06		0.083
	Age		**−0.40**	**0.011**
	Sex		0.12	0.420
	BMI		0.17	0.275
	BMD		−0.27	0.083
Medial epiphyseal		**0.30**		**0.002**
	Δ	0.15		**0.006**
	Age		**−0.39**	**0.008**
	Sex		0.12	0.388
	BMI		0.19	0.186
	BMD		**-0.40**	**0.006**
Total metaphyseal		**0.27**		**0.004**
	Δ	0.12		**0.017**
	Age		**−0.35**	**0.019**
	Sex		0.12	0.416
	BMI		0.15	0.302
	BMD		**−0.35**	**0.017**

*BMI* body mass index, *BMD* bone mineral density, *WOMAC* Western Ontario and McMaster Universities Arthritis Index

Significant values of *R* ^*2*^, Δ, and β are in bold

## Discussion

Our regression models suggested that tibial epiphyseal and metaphyseal BMD independently explained variance in total pain in patients with OA prior to TKR, whereby patients with lower BMD tended to have higher levels of pain. Regionally, our models indicated that medial epiphyseal BMD was a significant predictor of total OA-related pain, again whereby lower BMD was associated with higher levels of pain. These findings suggest that there may be potentially overlooked characteristics in proximal tibial BMD, such as trabecular BMD, which may have a role in the pathogenesis of OA-related pain.

The study findings support our previous research (using the same cohort), which investigated links between OA-related nocturnal pain and subchondral cortical and subchondral trabecular bone near the subchondral surface (0–10 mm from the surface). This previous study found a (nonsignificant) trend toward low medial BMD [13] in patients with severe nocturnal pain, which is in agreement with the study findings of low medial epiphyseal and total epiphyseal and metaphyseal BMD in patients with high levels of pain. We [13], and others [30], however, have questioned whether our previously observed trend toward low medial BMD was due to the presence of cysts or diminished bone architecture and/or mineralization. Subsequent follow-up analyses indicated that both cysts and BMD were independently associated with pain [36]. The novelty of this study was that we focused our analyses in epiphyseal and metaphyseal trabecular regions largely void of cysts to determine any potential independent associations between BMD and pain.

Of note, the study findings both support and contrast with the previous study which also identified high lateral focal BMD in the subchondral trabecular region (2.5–10 mm below the surface) in patients with severe nocturnal pain [13]. High lateral focal BMD may be explained by the presence of BMLs, chondro-protection, or altered loading. First, prior research in this cohort identified a positive association between nocturnal pain and BMLs [37]. Given that BMLs have higher local BMD than surrounding bone tissue [38], a positive association between nocturnal pain and BMD is foreseeable. Future research needs to evaluate whether high focal BMD measurements exactly overlay the BML locations. Second, high lateral focal BMD may be a consequence of chondro-protection developed via low trabecular bone density. To explain, recent finite element (FE) simulations indicated that reduced proximal tibial trabecular bone density results in lower subchondral bone stiffness [17] and lower cartilage stresses [39], the latter presumably due to improved congruence between articulations [40]. As many of the study participants had evidence of medial OA, low trabecular BMD may be a physiologic response to lessen medial cartilage stress. At the same time, this chondro-protective process would also naturally transfer more load to the lateral compartment since the two compartments function in parallel. This altered loading should result in loading-induced adaptation; specifically higher lateral BMD near the subchondral surface to meet the mechanical demands of higher load transmission. Third, many of the study participants with evidence of medial OA may be self-altering their knee kinematics and stance to off-load the medial compartment, with the aim of alleviating joint pain. This altered loading could result in loading-induced adaptation with higher lateral BMD and lower medial BMD [41]. Fourth, as higher BMD appears to be focused in subchondral regions (<10 mm from the tibial surface) [13], joint load may be primarily transferred through the subchondral cortical endplate and subchondral trabecular bone to the peripheral cortex, off-loading epiphyseal and metaphyseal trabecular bone, thus explaining lower BMD in these regions. However, this explanation warrants further research given that we did not find association between pain and alignment [36]. Studies using subject-specific FE modeling are needed to investigate load transmission and subchondral bone stiffness at different stages of pain severity and disease progression.

In this study we report a significant association between age and pain assessed by WOMAC, whereby older participants reported lower pain. Specifically, younger male patients reported higher WOMAC pain scores (Additional file 2: Table S2). We recommend further analysis in larger longitudinal studies to evaluate if this finding is unique to this sample or if this is more widespread within patients with OA. It is also worthwhile noting that we report no associations between age and BMD (Table 2, Additional files 1, 2 and 3: Tables S1–S3). This is in agreement with previous OA research reporting no association between age and BMD [35] or age and bone volume fraction [42, 43]. Although there is consensus that bone loss is associated with normal aging [44], this association appears not to pertain to bone tissue within the joint in OA. In support of this, there was no collinearity concern between BMD and age in our models predicting variance in pain. To further explore these associations, we ran the analysis with BMD as the dependent variable, pain as the independent variable, and age, sex, and BMI as covariates. These models suggested pain to be an independent predictor of BMD (Additional file 4: Table S4).

This study has certain limitations. First, pain severity and assessment was based on the entire knee joint, including all joint surfaces (tibiofemoral and patellofemoral) and tissues (e.g., bone, menisci, and synovium), and it is uncertain if pain originated within the proximal tibial bony structure, other tissues, or a combination of tissues. Second, although OA severity was homogeneous across study participants, all were in late stages of OA and it may not be possible to apply our findings to patients in the early stages of OA. Third, our study sample size was small (n = 41). Further analysis with larger samples including healthy participants and participants with various stages of OA severity and pain, are needed to verify these preliminary study findings. Of note, our sample comprised participants with severe OA (primarily with KL scores of 3–4). This limited range constrained our ability to include it in the statistical model. Also, with a basic rule of a minimum of 10 events (or samples) per predictor [45], we were limited to four predictors (independent variables) in each model: one independent variable (BMD) and three covariates (age, sex, and BMI), and thus other known predictors of pain were not assessed or investigated (e.g., smoking/alcohol history [46], activity level [1], mental health status [47], and specific medications). Of note, we attempted to account for possible differences in physical activity (mechanical loading/unloading) through use of cortical BMD measures at the 66% tibial shaft site. Previous work has identified differences in tibial shaft cortical BMD between highly active individuals (e.g., sprinters, endurance runners, triple-jumpers, high-jumpers, and hurdlers) and less active controls [48]. However, in this study, we did not note any associations between pain and tibial shaft cortical BMD, potentially indicating, at least to some degree, similar levels of activity and mechanical loading amongst study participants. Fourth, our 0.625-mm isotropic voxel size prevented assessment of trabecular microarchitecture and limited us to measurements of volumetric BMD. Accordingly, it is unclear if low BMD is due to trabecular thinning or wide trabecular spacing. For future research, it would be advantageous to investigate links between pain and trabecular microarchitecture with advanced texture analysis and smaller voxel sizes [8].

In this study we present statistically significant relationships as opposed to clinically significant relationships. As a statistically significant relationship does not measure the clinical effect of a result [49], it is important to consider the clinical effect that changes in epiphyseal or metaphyseal BMD may have on OA-related knee pain. According to Angst et al. [50], the minimal clinically important difference for OA-related pain is a change in WOMAC score greater than 6% of its maximum value (which is 20 for WOMAC). In other words, a change in pain will not be perceived unless the WOMAC score changes by 1.2 points. With this in mind, we can identify the BMD change that will correspond with a 1.2-point change in pain. Based on our model, a 44 g/cm^3^ reduction in epiphyseal or metaphyseal BMD will be marked by a perceived change in pain status. Assuming an average BMD of 100 g/cm^3^ for epiphyseal and metaphyseal bone, this would equate with ~ 50% change in density. Accordingly, a rational therapeutic approach would be to monitor bone while simultaneously striving to maintain bone and limit bone loss. Density changes in these regions could be monitored using QCT, dual-energy x-ray absorptiometry (DXA) or radiography. With regards to maintaining bone, potentially, this could be achieved through exercise interventions or pharmacological therapies. Our preliminary findings may also be clinically important for TKR preparation and planning. Patients with low preoperative BMD have been shown to be at higher risk of implant failure by loosening or migration [51], higher risk of revision surgery [52], and risk of failure following revision procedures [52]. Current tibial implant design components typically include a single central post, which is inserted through the tibial epiphysis and extends into the tibial shaft. Based on our findings, there may be low quantities of bone stock in individuals with higher levels of OA-related pain, potentially placing them at risk of inadequate osseo-integration and implant fixation [53] and possibly implant loosening [54]. As there is an expected normal decrease in tibial BMD during healing [55], reduced amounts of tibial epiphyseal bony support structure prior to TKR could compromise implant fixation and success in the early stages, potentially compromising long-term implant success. It may be beneficial to use imaging and complementary image-processing techniques to evaluate preoperative bone density, especially in the commonly overlooked tibial epiphyseal and metaphyseal regions, to compliment customized surgical approaches in patients with higher levels of pain.

## Conclusions

In our study, low tibial epiphyseal and metaphyseal BMD, and low medial epiphyseal BMD, was associated with OA-related pain in patients with severe OA prior to TKR. This study suggests that there may be overlooked characteristics within trabecular bone that may be related to the pathogenesis of OA-related pain in patients with severe OA. These preliminary findings from current and previous studies [13] may be valuable in guiding outcome selection in OA studies addressing subchondral bone and pain, particularly in determining regions of interest of the proximal tibia for potential epidemiological studies.

## Additional files

Additional file 1: Table S1. Coefficients (*r*) with 95% confidence intervals for correlation between all model variables for all included participants (*n* = 41). Significant associations are in bold. (DOCX 12 kb)

Additional file 2: Table S2. Coefficients (*r*) with 95% confidence intervals for correlation between all model variables for included male participants (*n* = 17). Significant associations are in bold. (DOCX 12 kb)

Additional file 3: Table S3. Coefficients (*r*) with 95% confidence intervals for correlation between all model variables for included female participants (*n* = 24). Significant associations are in bold. (DOCX 12 kb)

Additional file 4: Table S4. Adjusted coefficients of determination (*R* ^*2*^) and standardized beta coefficients (β) of the base model I (age, sex, and BMI) and base model II (age, sex, BMI and WOMAC pain) to predict variance in bone mineral density (BMD) at the total and regional proximal tibia. Significant *R* ^*2*^ and β values are in bold; *p* values are in parentheses. (DOCX 13 kb)

## Abbreviations

BMD: Bone mineral density
BML: Bone marrow lesion
CT: Computed tomography
CV%: Coefficients of variation
DXA: Dual-energy x-ray absorptiometry
FE: Finite element
HU: Hounsfield units
JSN: Joint space narrowing
KL: Kelgren-Lawrence score
OA: Osteoarthritis
QCT: Quantitative computed tomography
TKR: Total knee replacement
VIF: Variance inflation factor
WOMAC: Western Ontario and McMaster Universities Arthritis Index
