# Supplementary material for: Proximal tibial trabecular bone mineral density is related to pain in patients with osteoarthritis
Source: Arthritis Res Ther. 2017 Sep 12;19:200. doi: 10.1186/s13075-017-1415-9 (PMC5596910; doi:10.1186/s13075-017-1415-9)
Supplement: Supplementary file 1 — Coefficients (r) with 95% confidence intervals for correlation between all model variables for all included participants (n = 41). Significant associations are in bold. (DOCX 12 kb) [file 13075_2017_1415_MOESM1_ESM.docx]

Additional file

Table S1. Correlation coefficients (*r*), with 95% confidence intervals, between all model variables for all included participants (n=41). Significant associations are bolded.

|  | Age | BMI | Total WOMAC Pain | Total Epiphyseal BMD | Lateral Epiphyseal BMD | Medial Epiphyseal BMD | Total Metaphyseal BMD |
| --- | --- | --- | --- | --- | --- | --- | --- |
| Age | 1 | -0.30 (-0.61 to 0.01)  *p=*0.058 | -0.43* (-0.72 to -0.14)  *p=*0.005 | -0.06 (-0.38 to 0.26)  *p=*0.715 | -0.02 (-0.35 to 0.30)  *p=*0.881 | -0.02 (-0.35 to 0.30)  *p=*0.890 | 0.11 (-0.21 to 0.43)  *p=*0.499 |
| BMI |  | 1 | 0.19 (-0.12 to 0.51)  *p=*0.225 | 0.23 (-0.09 to 0.54)  *p=*0.151 | 0.24 (-0.07 to 0.56)  *p=*0.127 | 0.22 (-0.10 to 0.54)  *p=*0.169 | 0.11 (-0.22 to 0.43)  *p=*0.514 |
| Total WOMAC Pain |  |  | 1 | **-0.34 (-0.64 to -0.03)**  ***p=*0.030** | -0.26 (-0.57 to 0.06)  *p=*0.108 | **-0.38 (-0.68 to -0.08)**  ***p=*0.015** | **-0.40 (-0.70 to -0.10)**  ***p=*0.009** |
| Total Epiphyseal BMD |  |  |  | 1 | **0.86 (0.69 to 1.00)**  ***p*<0.001** | **0.89 (0.75 to 1.00)**  ***p*<0.001** | **0.91 (0.77 to 1.00)**  ***p*<0.001** |
| Lateral Epiphyseal BMD |  |  |  |  | **1** | **0.60 (0.34 to 0.86)**  ***p<*0.001** | **0.74 (0.52 to 0.96)**  ***p*<0.001** |
| Medial Epiphyseal BMD |  |  |  |  |  | **1** | **0.84 (0.66 to 1.00)**  ***p*<0.001** |
| Total Metaphyseal BMD |  |  |  |  |  |  | 1 |
