# Supplementary material for: Proximal tibial trabecular bone mineral density is related to pain in patients with osteoarthritis
Source: Arthritis Res Ther. 2017 Sep 12;19:200. doi: 10.1186/s13075-017-1415-9 (PMC5596910; doi:10.1186/s13075-017-1415-9)
Supplement: Supplementary file 2 — Coefficients (r) with 95% confidence intervals for correlation between all model variables for included male participants (n = 17). Significant associations are in bold. (DOCX 12 kb) [file 13075_2017_1415_MOESM2_ESM.docx]

Table S2. Correlation coefficients (*r*), with 95% confidence intervals, between all model variables for included male participants (n=17). Significant associations are bolded.

|  | Age | BMI | Total WOMAC Pain | Total Epiphyseal BMD | Lateral Epiphyseal BMD | Medial Epiphyseal BMD | Total Metaphyseal BMD |
| --- | --- | --- | --- | --- | --- | --- | --- |
| Age | 1 | -0.28 (-0.52 to -0.16)  *p=*0.274 | -0.54* (-0.92 to -0.75)  *p=*0.024 | 0.14 (-0.30 to 0.51)  *p=*0.593 | 0.10 (-0.35 to 0.50)  *p=*0.709 | 0.15 (-0.31 to 0.53)  *p=*0.571 | 0.27 (-0.18 to 0.54)  *p=*0.297 |
| BMI |  | 1 | -0.02 (-0.08 to 0.76)  *p=*0.954 | 0.13 (-0.49 to 0.78)  *p=*0.631 | 0.07 (-0.59 to 0.74)  *p=*0.802 | 0.14 (-0.49 to 0.82)  *p=*0.595 | 0.10 (-0.47 to 0.68)  *p=*0.697 |
| Total WOMAC Pain |  |  | 1 | **-0.50 (-0.80 to -0.02)**  ***p=*0.040** | -0.35 (-0.74 to 0.14)  *p=*0.167 | **-0.51 (-0.83 to -0.03)**  ***p=*0.035** | **-0.66 (-0.79 to -0.18)**  ***p=*0.004** |
| Total Epiphyseal BMD |  |  |  | 1 | **0.91 (0.70 to 1.00)**  ***p*<0.001** | **0.91 (0.70 to 1.00)**  ***p*<0.001** | **0.90 (0.59 to 1.00)**  ***p*<0.001** |
| Lateral Epiphyseal BMD |  |  |  |  | 1 | **0.69 (0.29 to 1.00)**  ***p=*0.002** | **0.78 (0.37 to 0.97)**  ***p*<0.001** |
| Medial Epiphyseal BMD |  |  |  |  |  | 1 | **0.80 (0.42 to 0.99)**  ***p*<0.001** |
| Total Metaphyseal BMD |  |  |  |  |  |  | 1 |
