# Supplementary material for: Proximal tibial trabecular bone mineral density is related to pain in patients with osteoarthritis
Source: Arthritis Res Ther. 2017 Sep 12;19:200. doi: 10.1186/s13075-017-1415-9 (PMC5596910; doi:10.1186/s13075-017-1415-9)
Supplement: Supplementary file 3 — Coefficients (r) with 95% confidence intervals for correlation between all model variables for included female participants (n = 24). Significant associations are in bold. (DOCX 12 kb) [file 13075_2017_1415_MOESM3_ESM.docx]

Table S3. Correlation coefficients (*r*), with 95% confidence intervals, between all model variables for included female participants (n=24). Significant associations are bolded.

|  | Age | BMI | Total WOMAC Pain | Total Epiphyseal BMD | Lateral Epiphyseal BMD | Medial Epiphyseal BMD | Total Metaphyseal BMD |  |
| --- | --- | --- | --- | --- | --- | --- | --- | --- |
| Age | 1 | -0.32 (-0.86 to 0.12)  *p=*0.133 | -0.38 (-0.83 to 0.30)  *p=*0.068 | -0.14 (-0.62 to 0.32)  *p=*0.509 | -0.07 (-0.54 to 0.40)  *p=*0.759 | -0.01 (-0.60 to 0.38)  *p=*0.658 | 0.07 (-0.46 to 0.59)  *p=*0.759 | |
| BMI |  | 1 | 0.34 (-0.07 to 0.68)  *p=*0.104 | 0.17 (-0.25 to 0.56)  *p=*0.433 | 0.22 (-0.20 to 0.60)  *p=*0.307 | 0.19 (-0.24 to 0.59)  *p=*0.387 | 0.04 (-0.40 to 0.48)  *p=*0.859 | |
| Total WOMAC Pain |  |  | 1 | -0.23 (-0.70 to 0.21)  *p=*0.289 | -0.16 (-0.61 to 0.28)  *p=*0.443 | -0.29 (-0.75 to 0.15)  *p=*0.174 | -0.28 (-0.78 to 0.16)  *p=*0.183 | |
| Total Epiphyseal BMD |  |  |  | 1 | **0.82 (0.56 to 1.00)**  ***p*<0.001** | **0.89 (0.71 to 1.00)**  ***p*<0.001** | **0.91 (0.79 to 1.00)**  ***p*<0.001** | |
| Lateral Epiphyseal BMD |  |  |  |  | 1 | **0.53 (0.16 to 0.94)**  ***p=*0.008** | **0.71 (0.43 to 1.00)**  ***p*<0.001** | |
| Medial Epiphyseal BMD |  |  |  |  |  | 1 | **0.84 (0.62 to 1.00)**  ***p*<0.001** | |
| Total Metaphyseal BMD |  |  |  |  |  |  | 1 | |
