# Supplementary material for: Proximal tibial trabecular bone mineral density is related to pain in patients with osteoarthritis
Source: Arthritis Res Ther. 2017 Sep 12;19:200. doi: 10.1186/s13075-017-1415-9 (PMC5596910; doi:10.1186/s13075-017-1415-9)
Supplement: Supplementary file 4 — Adjusted coefficients of determination (R 2) and standardized beta coefficients (β) of the base model I (age, sex, and BMI) and base model II (age, sex, BMI and WOMAC pain) to predict variance in bone mineral density (BMD) at the total and regional proximal tibia. Significant R 2 and β values are in bold; p values are in parentheses. (DOCX 13 kb) [file 13075_2017_1415_MOESM4_ESM.docx]

Table S4. Adjusted coefficients of determination (*R^2^*) and standardized beta coefficients (β) of the base model I (age, sex, and BMI) and base model II (age, sex, BMI and WOMAC pain) to predict variance in bone mineral density (BMD) at the total and regional proximal tibia. Significant *R^2^*, and β are bolded; *p*-values in parentheses.

|  | Total epiphyseal BMD | | Lateral epiphyseal BMD | | Medial epiphyseal BMD | | Total metaphyseal BMD | |
| --- | --- | --- | --- | --- | --- | --- | --- | --- |
| Base Model I | *R^2^* (*p*) | β (*p*) | *R^2^* | β (*p*) | *R^2^* | β (*p*) | *R^2^* | β (*p*) |
|  | 0.14 (0.134) |  | 0.12 (0.173) |  | 0.08 (0.374) |  | 0.08 (0.406) |  |
| Age |  | 0.01 (0.975) |  | 0.05 (0.765) |  | 0.04 (0.790) |  | 0.15 (0.371) |
| Sex |  | -0.30 (0.062) |  | -0.26 (0.111) |  | -0.18 (0.282) |  | -0.21 (0.202) |
| BMI |  | 0.15 (0.356) |  | 0.19 (0.256) |  | 0.19 (0.277) |  | 0.10 (0.374) |
|  |  |  |  |  |  |  |  |  |
| Base Model II |  |  |  |  |  |  |  |  |
|  | **0.27 (0.018)** |  | 0.20 (0.090) |  | **0.26 (0.028)** |  | 0.21 (0.067) |  |
| Age |  | -0.17 (0.316) |  | -0.08 (0.665) |  | -0.15 (0.372) |  | -0.02 (0.902) |
| Sex |  | -0.22 (0.146) |  | -0.20 (0.210) |  | -0.09 (0.574) |  | -0.13 (0.406) |
| BMI |  | 0.20 (0.195) |  | 0.23 (0.169) |  | 0.25 (0.126) |  | 0.15 (0.367) |
| WOMAC pain |  | **-0.42 (0.013)** |  | -0.30 (0.083) |  | **-0.48 (0.006)** |  | **-0.42 (0.017)** |
